# Analysis of Determinants of Dietary Iodine Intake of Adolescents from Northern Regions of Poland: Coastal Areas and Lake Districts

**DOI:** 10.3390/nu17243813

**Published:** 2025-12-05

**Authors:** Katarzyna Lachowicz, Małgorzata Stachoń

**Affiliations:** Department of Dietetics, Institute of Human Nutrition Sciences, Warsaw University of Life Sciences (SGGW-WULS), 159c Nowoursynowska Street, 02-776 Warsaw, Poland; malgorzata_stachon@sggw.edu.pl

**Keywords:** iodine, iodized salt, natural food, northern Poland, post-primary students

## Abstract

**Background/Objectives:** Iodine plays a central role in thyroid hormone synthesis and neurodevelopment. Its deficiency and excessive intake have been identified as risk factors for thyroid diseases and their consequences. The objective of the study was to analyze dietary iodine intake (dIi) and the factors that determine its intake among post-primary school students from northern Poland, specifically those from coastal areas and lake districts. **Methods:** The study was conducted on a sub-national sample of 3102 adolescents (1751 females and 1351 males) aged 14–20 years, recruited from schools located in the Northern (N) and North-Western (N-W) macroregions of Poland. Dietary iodine intake was assessed using the Iodine Dietary Intake Evaluation-Food Frequency Questionnaire. Based on the data obtained, the adequacy of the intake of this micronutrient was assessed. Statistical analysis was performed using the Shapiro-Wilk, U Mann-Whitney, and Kruskal-Wallis tests and Spearman’s correlation analysis. **Results:** The median dIi was 66.83 µg daily, including 53 µg from natural sources. This value was below the recommended dietary allowance of 150 µg and below the estimated average requirement of 95 µg of iodine in 85% and 68% of the study participants, respectively. Milk and dairy products provided the highest iodine intake (26.4%). Iodine-enriched salt (16.2%) also significantly impacted iodine intake. However, 60% of respondents did not use iodized salt. The median iodine levels from natural sources were found to be low (dairy products: 15.02 µg, fish and fish products: 2.38 µg, and eggs: 2.10 µg). Dietary iodine intake was significantly lower in adolescents from the N than N-W macroregion of Poland (median: 65.63 vs. µg daily, 74.2 *p* < 0.001). However, dIi did not depend on sex (*p* = 0.10), age (*p* = 0.80), school location (*p* = 0.80), body mass index classification (*p* = 0.76), or iodine supplementation (*p* = 0.90). **Conclusions:** The study results indicate that insufficient iodine intake among adolescents in northern Poland can be attributed to a limited intake of iodine from natural food sources. A pressing need exists to closely monitor iodine intake and status among Polish adolescents and to implement nutritional education, focusing on the role of iodine, potential risks associated with iodine deficiency, and dietary sources of iodine.

## 1. Introduction

Iodine is the second substrate, alongside the amino acid tyrosine, for the synthesis of thyroid hormones (THs), and the largest amount of this micronutrient is stored in the thyroid gland. The majority of the physiological effects of iodine are therefore mediated by THs, which regulate a variety of biochemical processes, including protein synthesis, carbohydrate and lipid metabolism. These hormones are essential for the proper development of the central nervous system during the fetal and infant periods. Furthermore, THs have been demonstrated to facilitate the optimal functioning of muscular, osseous, renal, cardiovascular, digestive, and reproductive systems. Furthermore, iodine is one of the earliest known and strongest antioxidants, exhibiting antibacterial, antiviral, antifungal, and anticancer properties [1,2,3].

Insufficient iodine intake, particularly during pregnancy and lactation, has been demonstrated to result in long-term cognitive impairment and delayed mental development in both childhood and adulthood. It has also been identified as a causative agent of a number of other iodine deficiency disorders (IDDs), including endemic goiter and hypothyroidism, reproductive disorders, reduced child survival, and varying degrees of growth and development disorders and metabolic disorders. Conversely, excessive iodine intake has been identified as a significant etiological factor in hyperthyroidism and autoimmune thyroid diseases, including Hashimoto’s disease and Graves’ disease. Therefore, monitoring iodine intake and status in children and adolescents is crucial [1,4,5,6,7,8].

The most important sources of iodine in the human diet are meat, milk, eggs, fish, and other animal products. However, these products contain too little iodine to cover the full daily requirement for this element. Additionally, consumption of natural sources of iodine is insufficient in most regions of the world, including Poland. Therefore, preventive measures have been introduced to combat iodine deficiency disorders. The most basic and recommended form of these measures is the iodization of table salt (either mandatory or voluntary). In some countries, this model has been supplemented by the iodization of other food products [3,5,9].

Numerous European countries, including Poland, have demonstrated the efficacy and safety of iodine prophylaxis. Eliminating iodine deficiency-related pathologies, such as endemic goiter and cretinism, is a significant milestone in public health. It is evident that Poland’s iodine policy, which involves the mandatory iodization of table salt (30 ± 10 mg of potassium iodide per kilogram of NaCl or 39 ± 13 mg of potassium iodate per kilogram of NaCl), has contributed to a notable reduction in the incidence of IDD. A review of the literature on urinary iodine concentration (UIC) in children aged 6–12 indicates that Poland, like most European countries, is currently classified as having a sufficient iodine intake [9,10,11,12,13].

However, it is important to acknowledge that the endeavor was not completely successful. Certain demographic groups, including children and school-age adolescents, remain susceptible to health complications arising from iodine deficiency. Some of these individuals have been observed to exhibit subclinical iodine deficiency, goiter, and subclinical hypothyroidism [3,12,13,14,15]. Large population studies have shown that thyroid dysfunction is a common diagnosis, affecting 2–6% of the general population worldwide [10,16]. According to data from the Central Statistical Office (CSO), in 2023, 8.1% of Poles reported having a thyroid condition diagnosed by a doctor [17]. In 2019, the incidence rate among adolescents and young adults (up to 29 years of age) averaged 4.4%. Surprisingly, the highest percentage of respondents declaring thyroid diseases was recorded in the coastal voivodeships (West Pomeranian: 7.6%; Pomeranian: 5.1%), i.e., areas with the highest iodine content in the air [18]. In excess of 20% of school-age children from the northern region of Poland exhibited UIC levels below 50 µg/l, indicative of moderate iodine deficiency [13]. This may be attributable to the avoidance of iodised salt and insufficient consumption of natural iodine-rich foods, such as fish and fish products, seafood, eggs, and dairy products. It is therefore evident that iodine deficiency continues to represent a significant public health problem worldwide, necessitating the identification and implementation of novel preventive solutions [3,4,19,20].

To our knowledge, no studies have assessed habitual iodine intake in adolescents at the sub-national or national level in Poland in recent years. Several previous studies conducted at the sub-national level have shown that a certain proportion of Polish teenagers aged 13–15, as well as young female soccer players, have an insufficient iodine intake [21,22]. Considering the aforementioned factors and the understanding that the IUC only reflects daily iodine intake, not habitual intake, an effort was made to assess the habitual iodine intake of adolescents enrolled in post-primary educational institutions in northern Poland, specifically those from coastal areas and lake districts. This evaluation encompassed the analysis of naturally occurring iodine sources and the use of iodized salt. The results obtained were related to the adequacy of iodine intake, and an analysis was made of the factors that may determine this intake. The daily iodine supply was stratified according to the macroregion of northern Poland, school location, sex, age, body mass index classification, and the use of iodine supplementation.

## 2. Materials and Methods

### 2.1. Ethical Aspects

The study was conducted at the Department of Dietetics, Warsaw University of Life Sciences (SGGW), in accordance with the Declaration of Helsinki. All procedures involving human participants were approved by the Ethics Committee of the Central Clinical Hospital of the Ministry of Interior and Administration in Warsaw (no. 2/2021).

Participation in the study was entirely voluntary. Informed, written consent for their children to participate in the study was provided by all students and/or parents or legal guardians of participating students. The questionnaire employed in this study was completed anonymously, thus precluding the collection of data that would permit identification of the respondent.

### 2.2. Research Design

Data used in this study were collected from 1 February 2022, to 31 March 2022.

The present cross-sectional study was conducted on a sample of students attending the following types of post-primary schools in northern Poland: first-degree vocational schools, general secondary schools, and technical schools. The list of schools included in the study was derived from the online National Register of Schools and Educational Institutions, the supervision of which is overseen by the Ministry of National Education [23]. The study considered two macroregions located in northern Poland: (1) the Northern Macroregion (N), encompassing three voivodeships: Pomerania, Kuyavian-Pomeranian, and Warmian-Masurian, and (2) the North-Western Macroregion (N-W), encompassing the following three voivodeships: West Pomeranian, Lubusz, and Greater Poland.

The selection of these two Polish macroregions was not accidental and was based on their specific geographic location: coastal regions (proximity to the Baltic Sea) and the three main Polish lake districts (Masurian, Pomeranian, and Greater Poland), which include numerous smaller lake districts. The West Pomeranian, Pomeranian, and Warmia-Masurian voivodeships are coastal voivodeships, but are also located within the Pomeranian Lake District (West Pomeranian and Pomeranian Voivodeships) and the Masurian Lake District (Warmia-Masurian Voivodeship). The Lubusz and Wielkopolskie Voivodeships are located in the Wielkopolska Lake District. The Kuyavian-Pomeranian Voivodeship, on the other hand, is situated at the intersection of the three main Polish lake districts mentioned above.

The selection process of schools for the study took into account the division of Poland’s territory into seven macroregions, within which sixteen voivodeships are grouped as basic administrative units (NUTS 1 units in the 2021 statistical division of Poland [24]). The delineation of this division was informed by a range of geographical, historical, cultural, and economic factors. Moreover, each voivodeship is further subdivided into subordinate administrative units, known as poviats.

The stratified random sampling of schools method was used to select study participants, with the aim of obtaining a representative sample from the two macroregions mentioned above: the North and North-Western. The sample selection procedure in this study was consistent with that used in another study conducted in Poland [25,26,27]. This selection was conducted in two stages: (1) poviats were drawn from the voivodeships, and then (2) schools were drawn from the poviats. In the first stage, stratified selection of poviats was conducted for two macroregions encompassing six voivodeships, i.e., 10 poviats were randomly selected from each voivodeship (this resulted in a total of 60 districts). In the second stage, 10 post-primary schools were randomly selected from all the selected poviats (resulting in 600 post-primary schools). Additionally, if there were insufficient schools in the selected district, schools from neighboring poviats were selected. The procedure described above allowed us to invite a total of 600 post-primary schools to voluntarily participate in the study. This invitation was sent via email to the principals of these randomly selected schools. Principals were informed of the study objectives and protocol, and those who agreed to participate were also provided with an electronic link to the questionnaire created for this study. Principals who received the electronic link to the questionnaire were asked to deliver it to students at their school, along with information about the study’s aims and objectives. Participation in the study was entirely voluntary for both principals and students. Furthermore, all parents or legal guardians of participating students provided informed, written consent for their children to participate in the study. The questionnaire was completed completely anonymously, making it impossible to collect data that would identify the respondent.

Participating students had the opportunity to complete the questionnaire at school during class, which allowed for supervision and support from teachers designated by the school principal. These designated teachers ensured that the responses students entered into the questionnaire were factually accurate. They were also available to clarify any concerns students raised and answer any questions they had. The questionnaire was completed and returned by 3158 students from 26 post-primary schools whose principals agreed to participate in the study.

### 2.3. Study Participants

We adopted the following inclusion criteria for the study:

- students had to be enrolled in a school whose principal had agreed to participate in the study,
- age from 14 to 20 years (normal age for this level of education in Poland),
- informed consent from participants and their parents or legal guardians to participate in the study.

We also adopted the following exclusion criteria for the study:

- pregnancy and/or lactation in the case of female students,
- incomplete questionnaire and unreliable data contained in the questionnaire (e.g., regarding number of servings of consumed foods and/or food groups, body height, weight). Both authors independently reviewed and verified the completeness and reliability of the data entered by the study participants in each questionnaire. Questionnaires that were incomplete and/or contained unreliable data were excluded from further analysis.

The study’s primary objective was to reach and examine the general population of adolescents from post-primary schools in two northern macroregions of Poland (Northern and North-Western), as well as to assess dietary iodine intake and analyze the factors determining this intake. Therefore, no aspect of the health status of the study participants was used as an exclusion criterion. No questions were asked about comorbidities or medications that could potentially influence iodine intake or absorption from the diet.

Taking into account the inclusion and exclusion criteria adopted for the study, a group of 3102 students from 26 post-primary schools in two northern Polish macroregions was selected for the study.

**Figure 1:**
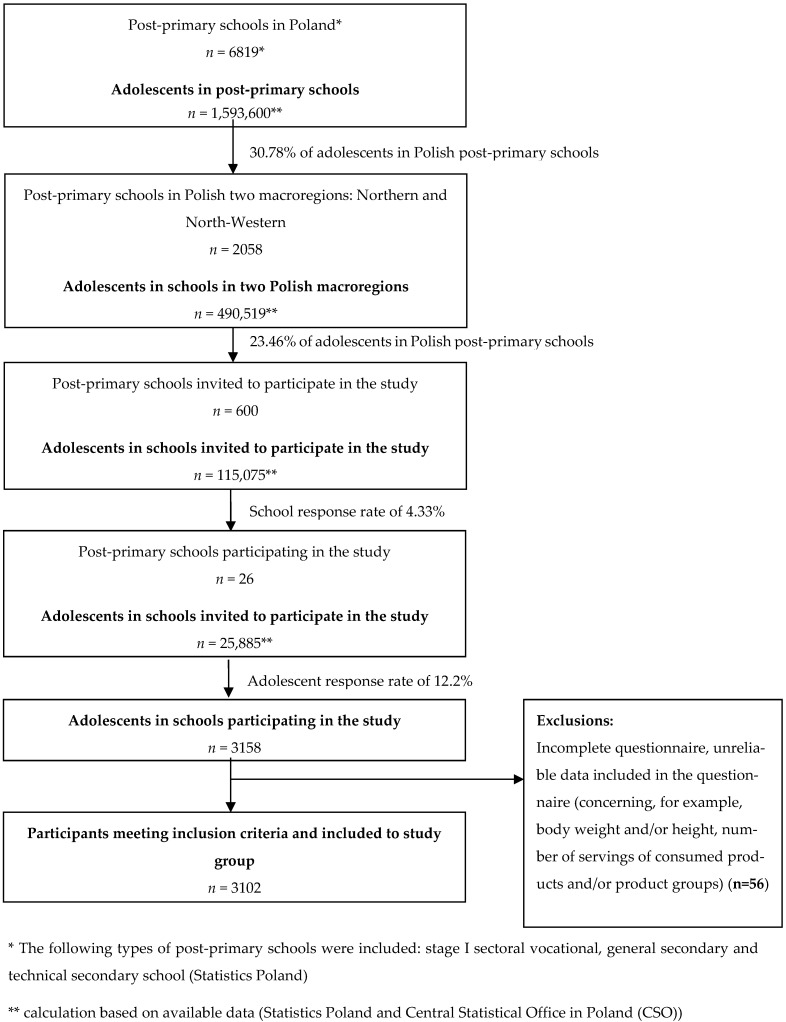
Procedure for sampling and recruitment to the research group.

Figure 1 presents the procedure for participant selection and recruitment to the study group.

### 2.4. Questionnaire

The data presented in this study were collected by means of the method of computer-assisted online interview [28].

Questions about gender, age, and the name of the school in a given voivodeship and district, included in the questionnaire used in this study, served to verify the inclusion and exclusion criteria.

Study participants who completed the questionnaire declared their use of supplements and/or medications containing iodine, i.e., the answer was “yes” or “no”.

The key element of the questionnaire used in this study was the Iodine Dietary Intake Evaluation-Food Frequency Questionnaire (IODINE-FFQ), developed for use in the Polish population to assess iodine intake from food. The IODINE-FFQ was developed and previously validated in a group of young Polish women [29]. This instrument has been included in the Register of Validated Brief Dietary Assessment Instruments by the National Institutes of Health (NIH)—National Cancer Institute of the United States of America [30]. The IODINE-FFQ is based on the assessment of food consumption frequency, taking into account only food products that are sources of iodine in amounts of at least 0.01 μg/100 g. These products are divided into thirteen groups: (1) milk and dairy products, (2) eggs, (3) meat, (4) fish, (5) fats, (6) cereal products, (7) vegetables, (8) legumes, (9) potatoes, (10) fruit, (11) nuts and seeds, (12) beverages, and (13) other (this group includes, among others, iodized salt) [29]. The Polish Tables of Food Composition, developed based on the reference chemical analysis of food products available on the Polish market [31], were used to select these food products. The IODINE-FFQ questionnaire contains questions regarding the consumption of specific food products in the year preceding the study, regardless of the season. In open-ended questions, study participants reported the number of servings of consumed foods, as well as the number of products added to their meals monthly or weekly (depending on the product). Importantly, participants had the option of indicating the number of servings not only as whole numbers but also as fractions [29]. The Polish Atlas of Portion Sizes of Food Products and Dishes [32] was used to determine portion sizes.

The data collected in the questionnaire for each participant allowed for the calculation of daily iodine intake as follows: (1) the total number of servings indicated by the participant was divided by seven or 30, for products consumed weekly or monthly, respectively; (2) iodine intake from each product was estimated using the following equation developed for the IODINE-FFQ: iodine intake [μg] = daily number of servings × iodine content per serving; (3) the total daily iodine intake from the diet was obtained by summing the iodine intake values from all thirteen food groups included.

The procedure delineated above enabled the calculation of the total iodine intake in the diet, as well as the iodine intake from individual product groups, including (1) fish and fish products (GR1: carp, eel, perch, pike, trout (rainbow and brown), sardine, sole, herring, flounder, salmon, mackerel, tuna, halibut, plaice, pollock, cod; GR2: smoked eel and other smoked fish (mackerel, salmon, herring); GR3: canned fish products (sprats and sardines in tomatoes and in oil, marinated herring and tuna) and herring in cream); (2) dairy products; (3) eggs; (4) meat and meat products; (5) cereal products; (6) vegetables, legumes, potatoes and fruit; (7) nuts and seeds; (8) beverages; (9) salt; (10) other (fats, gelatin, and chocolate) [31].

The following values were utilised in order to assess total intake of iodine from dietary sources and estimate the risk of iodine deficiency and/or excess, and their possible consequences: (1) the Polish nutritional standard for recommended dietary allowance (RDA) of 150 μg/day [33]; (2) the Polish nutritional standard for the estimated average requirement (EAR) of 95 μg/day [33]; (3) the Polish nutritional standard for the tolerable upper intake level (UL) of: 450 μg/day for individuals aged 11–14, 500 μg/day for individuals aged 15–17, and 600 μg/day for individuals aged ≥ 18 [33]; (4) marginal iodine intake below 50 μg/day [34]; (5) the intake protective against the development of goiter and hypothyroidism: the daily iodine intake per kilogram of body weight declared by the study participants [35]; (6) the low iodine intake 50–100 μg/day [34]; (7) the adequate and safe iodine intake i.e., between EAR and UL [36].

Furthermore, in order to assess salt intake as the primary source of iodine in the diet, a value of 5 g (equivalent to a teaspoon) was utilised, which represents the maximum daily intake of salt added to food and contained in food products [37,38].

The questionnaire used in this study included questions regarding current body weight (in kilograms) and height (in meters). Based on these self-reported data, the body mass index (BMI) was calculated using the Quetelet equation (body weight/height^2^). The OLAF program (“Development of the Reference Range of Blood Pressure for the Population of Children and Adolescents in Poland—PL0080 OLAF”) [39], based on Polish reference growth curves adjusted for gender and age [40], was used to assess body weight based on the calculated BMI in the 14–18 year old age group. In adult study participants (19–20 years old), the World Health Organization [41] classification was used to assess body weight based on calculated BMI.

### 2.5. Statistical Analysis

Statistica, version 13.3 (TIBCO Software Inc., San Ramon, CA, USA) was used to statistically analyze the study results.

The Shapiro-Wilk test was used to verify the normality of the data distribution.

Several statistical analyses were used to compare subgroups, including the Mann-Whitney U and Kruskal-Wallis tests, and the Spearman rank coefficient was used to analyze correlations due to nonparametric distributions.

The significance level was set at *p* ≤ 0.05.

For the purpose of subsequent analyses between subgroups, the study participants were divided into the following subgroups based on:

(1) The macroregion in which the school attended by the study participants was located, defined based on the macroregion categories adopted by the Central Statistical Office: North-Western and North [24];
(2) Location of the school attended by study participants: countryside, small city (under 20,000 inhabitants), medium city (between 20,000 and 100,000 inhabitants), and big city (over 100,000 inhabitants);
(3) Age: minors (from 14 to 17 years) and young adults (from 18 to 20 years);
(4) BMI categories: underweight, normal weight, overweight, and obesity–for adults, standard WHO cut-off values were used: below 18.5 kg/m^2^ for underweight, from 18.5 to 24.9 kg/m^2^ for normal weight, from 25 to 29.9 kg/m^2^ for overweight, and above 30 kg/m^2^ for obesity [41]. For minors, Polish height cut-off values were used [39]: below the 5th percentile for underweight, from the 5th to 85th percentile for normal weight, from the 85th to 95th percentile for overweight, and above the 95th percentile for obesity;
(5) Declaration of taking iodine-containing supplements and/or medications: “no” or “yes”;
(6) Dietary sources of iodine: (a) fish and fish products (GR1: carp, eel, perch, pike, trout (rainbow and brown), sardine, sole, herring, flounder, salmon, mackerel, tuna, halibut, plaice, pollock, cod; GR2: smoked eel and other smoked fish (mackerel, salmon, herring); GR3: canned fish products (sprats and sardines in tomatoes and in oil, marinated herring and tuna) and herring in cream); as well as other food groups (b) dairy products; (c) eggs; (d) meat and meat products; (e) cereal products; (f) vegetables, legumes, potatoes and fruits; (g) nuts and seeds; (h) beverages; (i) salt; (j) others (fats, gelatin and chocolate).

## 3. Results

### 3.1. General Characteristics of the Participants

The characteristics of the research group of post-primary school students, stratified by macroregion of northern Poland, school location, sex, age, body mass index classification, and iodine supplementation use, are presented in Table 1.

The higher percentage of participants hail from the Northern macroregion (approximately 78%), and more than half of the study group were female adolescents. The largest number of respondents attended schools in medium-sized towns (65.2%), and the smallest number in rural areas (3%). Most participants were between 14 and 17 years of age (71%), with the remaining 29% being adults (18–20 years of age). Nearly 95% of participants did not take iodine supplements. Over 70% of participants in both age groups had a normal body weight, with an average BMI of 21.98 ± 3.88 kg/m^2^ (24.43 ± 3.67 kg/m^2^ in females and 22.71 ± 4.02 kg/m^2^ in males).

### 3.2. Habitual dIi and Sources of Iodine

On average, the median dIi from all food sources (including iodine-fortified salt) in the studied group of adolescents from northern Poland reached 66.83 µg, with a range of 0.30 µg to 1176.21 µg per day. The natural dietary sources of iodine provided an average of 53.00 µg per day of this micronutrient, with a range from 0.30 µg to 169.79 µg (Table 2). The total dIi was found to be lower than the recommended daily iodine intake of 150 µg in Poland [33], which was met by only 15% of the participants. Iodine intake below RDA was reported in 81% of adolescents from the N-W macroregion and in 86% from the N macroregion, and in 84% male and 86% female participants. It was shown that 83% of students taking supplements and 85% of those not taking iodine supplements had dietary iodine intake below RDA. In both age groups, 85% of individuals had iodine intake below the RDA. The observed dietary iodine intake below the RDA ranged from 83% of pupils attending schools located in big cities to 85.3% in medium-sized cities, and from 84.5% among overweight students to 85.5% among obese students (Figure S1 in Supplementary Materials).

**Figure 2:**
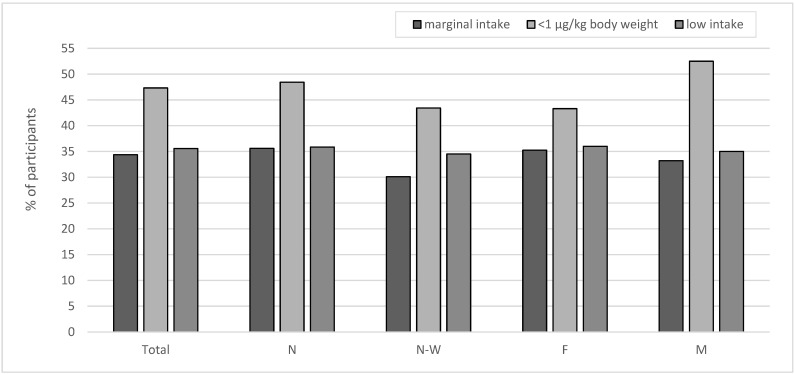
Percentage of adolescents with iodine intake: marginal (<50 µg/day), below 1 µg per kg body weight of participants and low (50–100 µg/day); N—Northern macroregion, N-W—North-Western macroregion, F—female, M—male.

**Figure 3:**
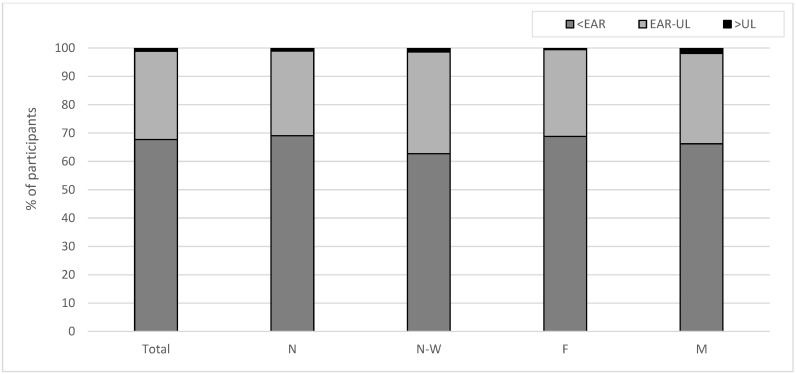
Percentage of adolescents with iodine intake: below estimated average requirement (<EAR; <95 µg/day), adequate and safe (i.e., between the EAR and UL) and excessive (i.e., above tolerable upper intake level; >UL: 450, 500 and 600 µg/day for individuals aged 14, 15–17 and ≥18, respectively); N—Northern macroregion, N-W—North-Western macroregion, F—female, M—male.

Moreover, it was observed that 67.7% of the participants (69.1% from N microregion and 62.3% from N-W microregion; 68.8% of females and 66.2% of males) had a dIi below 95 µg per day (lower than the EAR) and 47.3% (48.4% from N macroregion and 43.2% from N-W macroregion; 43.3% of females and 52.5% of males) had a dIi below 1 µg per kg body weight. Marginal iodine intake (<50 µg per day) was found in 34.4% of participants (35.6% from N macroregion and 30.1% from N-W macroregion; 35.24% of females and 33.23% of males), while low iodine intake (50–100 µg per day) concerned nearly 36% of the respondents (35.9% from N macroregion and 34.5% from N-W macroregion; 36% of females and 35% of males) (Figure 2). In less than ^1^/_3_ of the studied adolescent population (29.8% from the N macroregion and 35.9% from the N-W macroregion; 30.6% of females and 31.9% of males), the habitual iodine intake seems to be adequate and safe (i.e., between EAR and UL). Excessive iodine intake (>UL) was observed only in 1.16% (1.1% from the N macroregion and 1.4% from the N-W macroregion; 0.57% of females and 1.92% of males) of the subjects (Figure 3).

The following section presents data on dIi from various food groups by post-primary school students from northern Poland, who are the subject of the study. This data is presented in Table 2.

**Figure 4:**
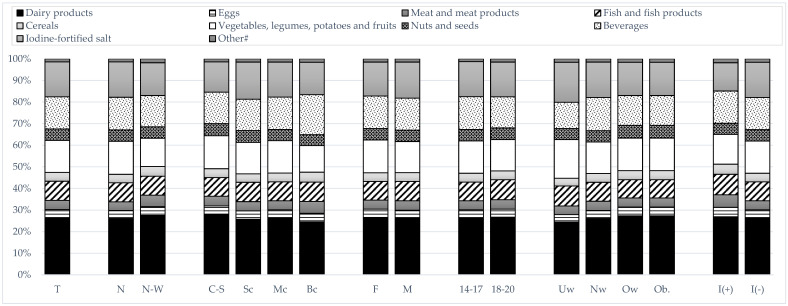
Dietary iodine sources in the subgroups of adolescents from northern Poland (T—total, macroregions: N—Northern, N-W—North-Western; location of the school: C-S—countryside, Sc—small city, Mc—medium city, Bc—big city; sex: F—female, M—male; age: 14–17 years old, 18–20 years old; Body Mass Index classification: Uw—underweight, Nw—normal weight, Ow—overweight, Ob.—obesity; iodine supplementation: I(+)—supplementing iodine, I(−)—not supplementing iodine); #—fats, chocolate, gelatin.

The median dIi was noted to be highest for dairy products (15.02 µg/day), followed by vegetables, legumes, potatoes and fruits (7.95 µg/day) and beverages (5.64 µg/day), while it was significantly lower for nuts and seeds (2.73 µg/day), fish and fish products (2.38 µg/day), meat and meat products (2.24 µg/day), cereals (2.10 µg/day) and eggs (2.01 µg/day), as well as it was lowest for fats, chocolate and gelatin (0.45 µg/day) and iodine-fortified salt (0.00 µg/day). The highest median dIi was documented for GR1, which included carp, eel, perch, trout, pike, sardine, sole, herring, flounder, salmon, mackerel, tuna, halibut, plaice, pollock, and cod among various species of fish and fish products. The following is a list of the percentage contribution of different food groups to the dIi: dairy products constituted 26.4% of the iodine, iodine-fortified salt 16.2%, beverages 14.9%, vegetables, legumes, potatoes and fruits 14.8%, fish and fish products 8.8%, nuts and seeds 5.3%, meat and meat products 4.3%, cereals 4.1%, and fats, chocolate, and gelatin 1.4%. A slightly higher percentage of dairy products in the iodine supply was noted for adolescents from the N-W macroregion, as well as for overweight and obese participants and students from rural schools. A marginally elevated proportion of iodine-fortified salt was observed in the diets of adolescents from the N macroregion, as well as in underweight and non-supplementing iodine-deficient participants and students from schools located in small cities (Figure 4).

**Figure 5:**
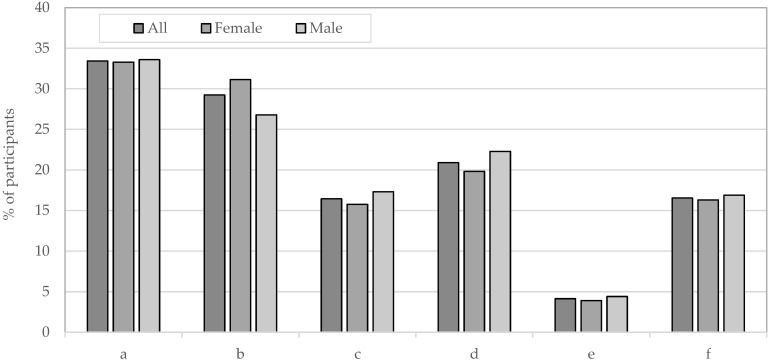
Percentage of adolescents from northern Poland consuming the following number of portions of fish and fish products: 0 (a), (0, 1) (b), ⟨1, 2) (c) and ≥2 (d), and not consuming: dairy products (e) and eggs (f).

As presented in Figure 5, the data indicate that 33.43% (33.29% female and 33.6% male) of the study’s participants reported zero consumption of fish and fish products. On the other hand, 20.89% of the students (19.82% female and 22.28% male) consumed at least two servings of fish per week. The remaining 29.24% (31.13% female and 26.79% male) consumed no more than one serving, and 16.44% (15.76% female and 17.32% male) consumed between one and two servings.

In addition, it was observed that some participants eliminated other products that are natural sources of iodine from their diet. Over 4% of the entire sample did not consume dairy products. This observation applied to 3.9% of females and 5.3% of males. A similar trend was observed in the consumption of eggs, where 16.5% of study participants, including 15.9% of females and 18.7% of males, declared that they did not consume eggs (Figure 5).

Furthermore, a higher percentage of young people from the N-W macroregion did not consume dairy products (5.3%) and eggs (18.7%), in comparison to those from the N macroregion (3.8% and 15.9%, respectively).

Despite iodized salt’s contribution to the study group’s iodine intake, 60% of respondents did not use iodine-fortified salt at all (see Figure S2 in the Supplementary Materials). The mean salt intake was 1.3 ± 2.17 g per day (range: 0.00–24.29 g), and on average, 4.1% of adolescents consumed more than 5 g of iodized table salt (see Figure S2 in the Supplementary Materials).

### 3.3. Determinants of dIi

Table 3 and Table S1 (in the Supplementary Materials) compare d-II consumption from different sources among subgroups of adolescents in two northern Polish macroregions.

The study demonstrated that pupils from the N-W macroregion exhibited higher total dIi (median: 74.20 µg per day) and iodine supply from dairy products (median: 18.46 µg per day) than pupils from the N macroregion (median: 65.63 and 14.34 µg per day, respectively; *p* < 0.001). There was regional variation in the contribution of other food groups to iodine provision, including meat and meat products (*p* < 0.001), cereals (*p* < 0.001), nuts and seeds (*p* = 0.002), and fats, chocolate, and gelatin (*p* < 0.001). See Table S1 in the Supplementary Materials and Figure 3 for details.

The findings demonstrated that the variables of sex, age, school location, body mass index category, and iodine supplementation were not significant contributors to the observed results (Table 3), suggesting a lack of dependency of total dIi on these factors.

As presented in Table S2 (Supplementary Materials), the location of the school determined iodine intake from meat and meat products (*p* = 0.010) as well as from fats, chocolate, and gelatin (*p* = 0.022). Pupils from big city schools consumed higher levels of iodine from meat and meat products than pupils from other schools. Furthermore, pupils from big city schools demonstrated higher levels of dIi compared to their peers from medium-sized cities.

As shown in Table S3 of the Supplementary Materials, sex was found to be a significant factor in determining iodine intake from meat and meat products (*p* = 0.002), as well as from GR3 fish and fish products (i.e., herring in a creamy sauce, pickled herring, and fish products in tins, *p* = 0.044). The contribution of these products as a source of iodine was higher among male adolescents.

The contribution of ten analyzed food groups as sources of iodine was similar in underage and full-grown adolescents, as well as in adolescents with normal and abnormal body weight (see Tables S4 and S5 in the Supplementary Materials). As presented in Table S6 (in the Supplementary Materials), the utilization of iodine supplements was found to be associated with increased iodine intake from eggs (*p* = 0.023), meat and meat products (*p* < 0.001), and from fats, chocolate, and gelatin (*p* = 0.0016). The analysis revealed that subjects who received iodine supplements demonstrated higher dIi values from these food groups compared to those who did not receive supplementation.

### 3.4. Correlations

In Table 4, the results of the analysis of the correlation between iodine intake from different food groups and total iodine intake, as well as between iodine-fortified salt intake and BMI and total iodine intake, are presented in a group of adolescents from northern Poland.

A significant positive correlation was demonstrated between total dIi and the intake of iodine from ten analyzed food groups. The strongest relationships were identified for iodine-fortified salt and dairy products. Total iodine intake (µg/day) was positively correlated with iodine-fortified salt intake (g/day).

There was no significant correlation observed between total dIi and BMI.

## 4. Discussion

The current study examined dietary iodine intake from various sources among a population of male and female adolescents attending schools in northern macroregions of Poland. The study found that the median dIi was low (66.83 µg daily from all food sources and 53 µg from natural sources only) and significantly lower than the EAR and RDA. Sixty-nine percent of respondents had a dIi below the EAR, and 95% had a dIi below the RDA. Adequate and safe iodine intake was observed in one-third of participants, while excessive iodine intake was noted in 1.16% of subjects. Dairy products provided the highest iodine intake, while fats, chocolate, and gelatin provided the least. Iodine-enriched salt, beverages, vegetables, legumes, potatoes, and fruits also significantly impacted iodine intake. Dietary iodine intake was significantly lower in adolescents from the N macroregion than in the N-W macroregion of Poland, which is characterized by greater socioeconomic development potential. On the other hand, the results showed that dIi was not dependent on sex, age, school location, body mass index classification, or iodine supplementation. Total dIi was strongly positively correlated with iodine from iodine-fortified salt and dairy products, but not with BMI.

Dietary iodine intake in the studied group of post-primary students from northern Poland was lower than in the other sub-national cross-sectional studies carried out in Poland on group of 13–15 years old boys from the sport schools (mean ± standard deviation: 186 ± 68 µg, range: 39.7–356 µg per day) [21] and on group of young (average age 21 ± 5 years) female professional soccer players (median: 86.37 µg, range: 6.53–236 µg per day) [22].

The results of other studies that examined adolescents aged 11–17 years and young adults aged 18–24 or 18–34 years included in the systematic review by Bath et al. (2022) also showed higher than in our study iodine intake among young participants from most European countries: Austria (median: 102–129 µg), Belgium (median: 111–160 µg), Denmark (median: 201 µg), Estonia (mean: 130 µg), France (mean: 134 µg), Germany (median: 73–94 µg), Iceland (mean: 116–169 µg), Ireland (median: 95–154 µg), Netherlands (median: 179–223 ug), Norway (median: 110–153 µg), Slovenia (mean: 181–205 µg), Spain (median: 87–105 µg) and United Kingdom (median: 105 µg) [42].

Additionally, higher iodine intake than that reported in our study was observed in populations studied in the following locations: Slovenian adolescents (mean: 189.7 μg) [43], Danish adolescents aged 15–17 (median: 155–223 μg) [44], Norwegian teenagers and young adults aged 16–24 (median: 109–129 μg) [45], Swedish adolescents aged 16–24 (160–170 μg) [46], three studies among German adolescents aged 13–17 (median: 74.1–112 μg) [47], adolescents aged 14–17 from Spain (90.1–104.6 μg) [48], children and adolescents aged 5–18 from Ireland (median: 94.8 μg) [49], and the Italian pediatric population up to 17 years of age (median: 109 μg) [50]. The average iodine intake was lower than in our study only among young adults (ages 19–34) in Lithuania (mean: 30 µg) and among adolescents and young adults in Turkey, especially females (mean: 55–57 µg) [42].

Almost all of the studies of males and females included in the above systematic review and the above-listed studies, unlike in our survey, observed higher iodine intake in male adolescents and young men than in female adolescents and young women [42,43,44,47,48,50]. The exceptions were a Slovenian study that reported higher iodine intake among female adolescents, as well as German and earlier Slovenian studies that, like our study, showed similar iodine intake among females and males [34,42,47].

In contrast to our studies in other European countries, lower iodine intake was found among younger adolescents compared to older adolescents and young adults when the studies were conducted in different age groups. Turkish surveys, like our study, found no effect of age on iodine intake [42].

A comparison of these data is complicated by the utilization of disparate instruments to evaluate iodine intake across the included studies. These instruments encompass 24-h recall, food diaries, and FFQ. FFQ has been observed to exhibit a tendency to overestimate intake, while the other methods have been shown to underestimate consumption [29].

Although adolescents in most European countries had higher iodine intake than those in northern Poland, intake was not sufficient or adequate in all these countries. In Austria and Germany, iodine intake below the EAR was reported in adolescents of both sexes. In Norway, Spain, and the United Kingdom, it was reported in female adolescents. In Lithuania and Turkey, it was reported in male and female adolescents. Conversely, iodine intake exceeding the recommended 150 µg (RDA) was observed in adolescents and young adults from Belgium, Denmark, the Netherlands, Sweden, and Slovenia, as well as in male adolescents and young men from Estonia, Iceland, and Ireland [34,42,43,46]. In our study, only 15% of adolescents followed these recommendations.

Nearly one-third of adolescents in northern Poland had an adequate iodine intake. This percentage was significantly lower than in other studies. A higher percentage of young Polish female soccer players (46%) [22] and school-age girls in Ireland (60%) [49] had an adequate iodine intake. In Norway and Sweden, adequate iodine intake was found in 58% and 77% of adolescents, respectively [45,46], while in Denmark, 89% of teenage girls and 97% of teenage boys had adequate intake [44]. An even higher percentage of female and male teenagers (98% and 99%, respectively) with adequate (>EAR) and safe (<UL) iodine intake was reported in the Netherlands. However, when considering only iodine from natural sources, this percentage decreased significantly, reaching levels lower than in our study (6% and 18% for females and males, respectively) [36].

The lower adequacy of iodine intake and low compliance with the recommended intake of this micronutrient indicate a higher risk of iodine deficiency among Polish adolescents than in other European countries. Furthermore, since more than 47% of surveyed adolescents were found to have an iodine intake below 1 µg per kilogram of body weight (with nearly 73% having marginally low intake), it can be assumed that the iodine supply is insufficient for normal thyroid function and thyroid hormone synthesis. Consequently, this group is at high risk for IDD, including hypothyroidism, goiter, cognitive function impairment, and delayed physical development [3,35]. A Slovenian study [34] found that a much smaller percentage of young people had marginal (3.3%) and low (20.3%) iodine intake compared to Polish adolescents from northern Poland (34.4% and 35.6%, respectively).

On average, 84% of adolescents in northern Poland’s iodine intake comes from naturally occurring iodine in food, while 16% comes from iodized salt. In other European countries, the proportion of iodine derived from naturally iodine-rich foods was lower (40–73%), while the proportion derived from iodized salt was higher (27–57%) [36,42,43,44,50]. This difference is due to the fact that 43% of the iodine came from salt added by food manufacturers, 90% of which came from bread prepared with iodized bakery salt, while only a few percent came from salt added during food preparation and seasoning. However, our study only considered salt added at the consumer’s discretion. Therefore, the amount of iodine from table salt added by consumers was similar in some studies [36,44], while in others the proportion of table salt was higher (37%) [43,50] than in our study.

The lower proportion of salt as a source of iodine in our study may be due to the young participants limiting their salt intake. Salt intake in our study was significantly lower (1.3 g) than in other studies (4.2–12.4 g per day) [47,50] and 60% adolescents did not use iodine-fortified salt at all. Another Polish study estimated salt intake from only salty snacks at 1.083 ± 0.09 g in a group of children and adolescents aged 10–18 [51]. Other studies have shown that 85% of adolescents use iodized salt [47]. However, it should be noted that controlling salt intake among teenagers is difficult because they usually don’t cook for themselves [52].

The main sources of iodine naturally present in the diets of adolescents from northern Poland were dairy products (26.4%), beverages (14.9%), and vegetables, legumes, potatoes, and fruits (14.8%), as well as fish and fish products (8.8%). Adolescents from other European countries also had a high iodine intake from dairy products, similar to our study results (30% in Germany, 32% in the Netherlands, 29% in France, and 26–27% in Denmark) [36,42,44].

A higher proportion of iodine intake came from dairy products among adolescents in Norway (56%) and the United Kingdom (51%) and school-age girls in Ireland (over 50%) [42,49] than among Polish adolescents. A lower iodine proportion comes from dairy products among adolescents in Belgium and the Netherlands, where cereal products accounted for a larger share. This may be because the salt used in bread production in Belgium and the Netherlands is iodized [42].

Adolescents from Poland and the Netherlands, as well as those from Poland and France, had similar contributions of iodine intake from beverages and fish, respectively [36,42]. Polish post-primary students had a higher iodine intake from fish than Belgian adolescents, but a lower intake than Norwegian teenagers. The lower iodine intake in the Polish youth diet compared to Norwegian teenagers may be due to a lower consumption of sea fish, which is high in some Nordic countries [42].

Similarly, the low proportion of eggs in the iodine intake of young people in northern Poland and other European countries [42] may be due to the limiting or elimination of eggs from their diets. Our survey found that 16% of post-primary students did not consume eggs. Such restrictions and eliminations may also apply to fish and fish products, as well as milk and dairy products. These restrictions significantly reduce iodine intake and increase the risk of iodine deficiency, as demonstrated by models that exclude specific food groups. In particular, eliminating dairy from one’s diet leads to inadequate iodine intake, especially among female adolescents [44]. Following a vegan or vegetarian diet, as well as other elimination diets, results in a significant reduction in iodine content and an even greater deficiency [45,46,53].

Habitual dietary iodine intake is a crucial yet understudied and insufficiently researched factor in determining iodine status [12]. The results suggest that consumption of iodine-rich foods, including iodized salt, is low in the pediatric population [50]. This could reduce the effectiveness of the national iodine deficiency prevention program, which has been evaluated as being among the best in Europe [54]. Despite preventive measures, iodine status has already declined significantly in some European countries [20,36], which may lead to iodine deficiency in the near future, especially in vulnerable populations. This was noted in the Krakow Declaration on Iodine, which was signed in 2018 [54]. Therefore, iodine intake assessment should be included in public health measures that evaluate the effectiveness of the current iodine prevention model in Poland and other European countries. Additionally, information and education campaigns aimed at increasing iodine intake should be an important element of iodine prevention. These aspects have already been highlighted in the objectives of the EU-funded project, EUthyroid, in which 27 European countries, including Poland, are participating [55]

In the studied population of adolescents attending schools in two macroregions of Poland, iodine intake was higher in the North-Western macroregion compared to the Northern macroregion, which was due, among other things, to higher consumption of the main dietary source of this element, i.e., dairy products. Iodine intake from other, less significant dietary sources (i.e., meat and meat products, eggs, cereals, nuts and seeds, and others) by adolescents was also higher in the N-W macroregion than in the N macroregion. However, iodine intake from other dietary sources of iodine (i.e., fish and fish products, vegetables, legumes, potatoes and fruits, beverages, and iodine-fortified salt) was similar among study participants.

The impact of the macroregion on iodine intake and differences in dairy consumption in both macroregions identified in the study is both interesting and intriguing. However, the interpretation of these findings is hindered by the paucity of data on regional disparities in food consumption in Poland, particularly among different age demographics. Despite the existence of data from the Polish Central Statistical Office concerning the monthly consumption of selected food products per person in households in individual provinces, these data do not differentiate consumption by age. Furthermore, the data demonstrate that the consumption of dairy products is comparable across both macroregions [56].

However, it is important to note that the primary regions responsible for the production of cow’s milk in Poland are the voivodeship of Mazovia (Mazovia Province Macroregion), Podlasie (Eastern Macroregion), and Greater Poland (North-Western Macroregion). Furthermore, an analysis of cow milk yield indicators in the provinces of the North-Western macroregion reveals higher values than those observed in the provinces of the Northern macroregion. It therefore appears that such regional conditions and diversity may have exerted a positive influence on the consumption of milk and dairy products by the study participants attending schools in the North-Western macroregion, in comparison to the lower consumption by participants attending schools in the Northern macroregion [57,58].

Regional differences in iodine intake were also demonstrated by Verkaik-Kloosterman et al. (2017) [36]: children aged 7–18 living in the western region of the Netherlands had a 6% higher iodine intake compared to children living in the southern region. The authors believe that this can be partly explained by regional differences in the consumption of milk and dairy products, one of the main dietary sources of this element: higher in the west and lower in the south.

Other data allow conclusions to be drawn about other factors influencing food consumption, including milk and dairy products, in individual macroregions of Poland. These are economic factors indicating certain differences within the N-W and N macroregions in terms of indicators such as unemployment rates, wage levels, and prices, but it is difficult to use them unequivocally to explain the differences in milk and dairy consumption, and thus iodine from these food sources, identified in this study [59,60].

It is possible that other factors, as indicated in the literature on the subject, such as the influence of family and friends, nutritional knowledge and level of education, eating habits and customs, and factors related to dairy products themselves (e.g., sensory characteristics, availability, convenience of consumption), determined the variation in the consumption of this food group and thus iodine by the young people surveyed [61,62,63,64,65].

A further group of foods with high iodine content, namely fish and fish products, contributes to a lesser extent to overall iodine intake. This is attributable to the comparatively lower popularity of fish and their lower consumption levels. A general downward trend in the consumption of fish and fish products has been observed in recent years in European Union countries, including Poland [66,67,68,69]. This downward trend is likely attributable to the multifaceted nature of fish and fish product consumption, which may also be factors limiting this consumption. The primary factors influencing fish and fish product consumption include: age and gender, socioeconomic status, geographical location (including proximity to areas with a high abundance of fish, such as coastal regions and lake districts), nutritional and culinary expertise (defined by the capacity to prepare palatable fish dishes and meals), price, sensory attributes, prevailing trends, and dietary habits (including familial practices within the domestic environment). It is important to note that fish and fish products possess highly distinctive sensory characteristics (taste, smell, appearance), which play a pivotal role in determining their consumption. Consequently, the decision-making process involved in the purchase and consumption of fish and fish products is intricate and necessitates numerous considerations. It appears that adolescents constitute a particularly sensitive population group, exhibiting vulnerability to the multifaceted influences of various factors in this domain, chiefly encompassing fashion trends disseminated via the Internet and peer pressure. Additionally, individual conditions that determine the selection of consumed food products are deemed to be a salient factor [26,70,71,72].

Furthermore, the consumption of fish in Poland is characterised by its occasional nature, being contingent on cultural and religious factors. The consumption of fish is known to increase during specific periods, such as the Christmas and Easter holidays, on Fridays for those observing religious fasting, and during the summer months due to the increased demand for fish-related products in restaurants and service establishments, particularly in coastal and lakeside areas [68,73]. Nutritional knowledge about the benefits of consuming this group of products is of rather minor importance.

It has been demonstrated that the presence of factors conducive to increased consumption of fish and fish products, mainly nutritional knowledge (including the benefits of consuming the recommended amount of fish) and proximity to coastal and lake areas (i.e., the availability of this product group), does not necessarily translate into increased consumption. According to data from the Central Statistical Office on households, this consumption does not differ between voivodeships [74]. Additionally, in the Utri-Khodadady and Głąbska (2023) study, knowledge about the benefits and safety of fish consumption among young people from areas far from the sea was higher than among those from areas located near the sea [69]. Therefore, the availability of fish and fish products associated with proximity to coastal and lake areas is a more complex factor determining their consumption.

### 4.1. The Study’s Strengths and Weaknesses

This study was conducted on a large sample of adolescents of both sexes from two macroregions of Poland located in the coastal region and the lake districts: the North and Northwest.

The FFQ questionnaire used in the study is highly relevant due to its validity and repeatability assessments, providing substantial data on iodine intake and its determinants in the Polish adolescent population. This is particularly important when formulating dietary strategies to optimise iodine intake.

A notable limitation of this study is the relatively low participation rate of schools, with only 26 of the 600 selected schools participating, which may introduce selection bias.

Another limitation of this study is that an indirect data collection method was used. Data were collected via an online questionnaire. Participants completed the questionnaire through self-assessment and self-report. This method, combined with the retrospective nature of the study, may have introduced recall bias, potentially influencing the accuracy and/or completeness of the dietary intake data presented in this study. Conversely, the utilisation of this method substantially expedited the dissemination of the invitation to participate in the study to a considerable number of individuals. Furthermore, as the collected data related to fish consumption, which is not usually eaten daily (but rather sporadically in Poland), using an alternative method (e.g., a 24-h dietary recall or a 3-day food diary) would not have produced reliable results. In addition, questionnaires that were incomplete and/or contained unreliable data, particularly regarding body weight, height, and the number of servings of products and product groups consumed, were excluded from further analysis. Excluding incomplete questionnaires may have affected the results, but probably to a lesser extent than if inappropriate questionnaires had not been rejected.

A potential limitation of this study, resulting from the fact that participants completed the questionnaire based on self-assessment and self-description, may have resulted in an underestimation of salt intake data. This may have been influenced by the fact that some participants (at least those who lived with their parents or legal guardians) did not prepare their own meals and therefore had no knowledge of the salt content of their food and dishes.

Another limitation of the study is that the questionnaire did not consider seasonal variations in food consumption. This can be attributed to the design of the tool. Subjects were asked about their consumption of specific food products in the year prior to the study, regardless of the season. Furthermore, no enquiries were made about the reasons for consuming or abstaining from fish and other foodstuffs. However, the most significant factor was the time it took participants to complete the questionnaire. Incorporating supplementary enquiries would have increased the time required to complete the questionnaire, which could have led to a reduced response rate.

It is important to note that participation in the study was entirely voluntary. Consequently, despite the sampling plan’s representative character, the collected data are not representative of the sample due to the insufficient response rate. However, it is important to note, given the aforementioned limitations, that the analysis was conducted on a substantial sample of over 3000 adolescents of both sexes from two selected macroregions of Poland.

### 4.2. Practical Applications and Directions for Future Research

The practical applications of the results of this study include: (1) identifying and indicating population groups most at risk of iodine deficiency; (2) identification of areas for seeking solutions to increase the consumption of products that are natural sources of iodine; (3) planning education for young people and their parents and/or legal guardians as part of school classes in accordance with the current core curriculum; (4) setting public health goals.

Iodine prophylaxis implemented in Poland, which includes measures to prevent iodine deficiency, is primarily aimed at counteracting psychosomatic developmental disorders, thyroid disease, and cardiovascular disease in children, adolescents, and adults. Nevertheless, in order to ensure the efficacy of preventive measures, it is essential to monitor their effectiveness and modify them as necessary in order to prevent both iodine deficiency and excess supply. The results obtained in this study constitute a significant element of this prophylaxis, specifically monitoring its effectiveness.

Consequently, future studies should examine the iodine intake from various sources in various population groups, with a particular focus on those at risk of iodine deficiency and/or excess. It is also crucial to strive for a consensus between the recommended salt restriction for the prevention of cardiovascular and kidney disease in adults, children, and adolescents, and ensuring iodine intake is consistent with current recommendations from authorised institutions. This issue is currently becoming increasingly important in light of the growing popularity of various plant-based diets and plant-based alternatives to animal products, among both adolescents and adults. The accelerated growth of the plant-based food market is a salient phenomenon that merits attention. This evolution may have the potential to reduce iodine intake from dietary sources, as plant-based products, as well as plant-based alternatives to animal products (including fish and dairy), are naturally low in iodine.

Milk and dairy products are the second most iodine-rich food products after salt. However, the consumption of these foods is subject to considerable variation and is susceptible to changes and trends in dietary habits. It is essential to recognize the heightened vulnerability of adolescents as a demographic, making them particularly susceptible to the influence of advertising, trends, and social media messages. A further group of foods with high iodine content, namely fish and fish products, contributes less to the overall iodine intake. This is due to the fact that fish is a less popular choice and its consumption is generally lower.

The development of effective strategies to encourage the youth population to increase their consumption of the aforementioned food groups (i.e., fish and fish products, and milk and dairy products) is imperative. Additionally, there is a need to adapt the range of products offered to align with consumer preferences, with a particular emphasis on their sensory attributes. It also seems beneficial to promote increased consumption of plant products naturally rich in iodine and to encourage the choice of iodine-enriched alternatives to animal products, where possible, in order to meet consumers’ iodine requirements.

## 5. Conclusions

In conclusion, the dietary iodine intake of post-primary school students in northern Poland was found to be low. This can be attributed to a limited intake of iodine from natural food sources, such as fish, fish products (although access to fish should be high in this area), eggs, dairy products, and iodine-enriched salt. The determinants of dietary iodine intake among the study participants were consumption of foods naturally rich in iodine, use of iodized table salt, and the macroregion of northern Poland. However, iodine intake was not associated with sex, age, school location, nutritional status, or the use of iodine supplements. There is an urgent need to closely monitor iodine habitual intake and status among Polish adolescents and implement targeted intervention measures within this population. These measures should include nutritional education that focuses on the role of iodine, the risks associated with iodine deficiency, and the dietary sources of iodine.

From a public health perspective, therefore, it is recommended that action be taken not only at the level of educational institutions and the adolescent environment, but also by local and national authorities. These measures may include routine monitoring of iodine policies, as well as assessment of levels of markers of iodine status in adolescents’ blood and urine. This would facilitate the early detection of deficiencies in high-risk groups, such as young people. This approach would allow for timely intervention, minimizing adverse effects and progression to more serious health consequences such as hypothyroidism, goiter, and cognitive impairment. Further population-based studies are needed to determine adolescents’ habitual total iodine intake, considering all sources of this micronutrient in relation to iodine status, and to assess the risk of iodine deficiency disorders.

## Figures and Tables

**Table 1:** Research group characteristics.

Variable			*n*	%
Total			3102	100
Macroregion of northern Poland	Northern		2418	77.95
	North-Western		684	22.05
Location of the school	Countryside	Total	342	11.02
		Northern	240	7.74
		North-Western	102	3.29
	Small city (<20,000)	Total	644	20.76
		Northern	488	14.44
		North-Western	196	6.32
	Medium city (20,000–100,000)	Total	2022	65.18
		Northern	1709	55.09
		North-Western	313	10.09
	Big city (>100,000)	Total	94	3.04
		Northern	21	0.68
		North-Western	73	2.35
Sex	Female		1751	56.45
	Male		1351	43.55
Age	14–17 years old		2195	70.76
	18–20 years old		907	29.24
Body Mass Index (BMI) classification	Underweight	All	156	5.03
		Female	115	3.71
		Male	41	1.32
		14–17 years old	89	2.87
		18–20 years old	67	2.17
	Normal weight	All	2178	70.21
		Female	1251	40.33
		Male	927	29.88
		14–17 years old	1565	50.45
		18–20 years old	613	19.76
	Overweight	All	465	14.99
		Female	236	7.61
		Male	229	7.38
		14–17 years old	316	10.19
		18–20 years old	149	4.80
	Obesity	All	303	9.77
		Female	149	4.80
		Male	154	4.97
		14–17 years old	225	7.25
		18–20 years old	78	2.51
Iodine supplementation	Yes		164	5.29
	No		2938	94.71

**Table 2:** Intake of iodine from different food groups in adolescents from northern Poland.

Iodine Source		Dietary Iodine Intake in µg Per Day	
		Mean ± Standard Deviation	Median (Min–Max) *
Total from all food sources ^1^		95.88 ± 105.36	66.83 (0.30–1176.21)
Total from natural sources		72.35 ± 84.63	53.00 (0.30–169.79)
Dairy products		21.91 ± 23.61	15.02 (0.00–283.20)
Eggs		2.75 ± 3.27	2.01 (0.00–26.86)
Meat and meat products		3.04 ± 2.97	2.24 (0.00–22.43)
Fish and fish products	Total	5.77 ± 9.61	2.38 (0.00–95.42)
	GR1	4.24 ± 7.74	1.62 (0.00–81.79)
	GR2	1.02 ± 2.36	0.00 (0.00–33.30)
	GR3	0.48 ± 1.18	0.00 (0.00–16.97)
Cereals		2.95 ± 2.90	2.10 (0.00–26.88)
Vegetables, legumes, potatoes and fruits		11.12 ± 11.98	7.95 (0.00–155.90)
Nuts and seeds		4.70 ± 7.63	2.73 (0.00–69.39)
Beverages		19.39 ± 68.47	5.64 (0.00–807.10)
Iodine-fortified salt		23.53 ± 49.74	0.00 (0.00–556.60)
Other #		0.72 ± 1.02	0.45 (0.00–12.71)

^1^ including fortification of household salt; GR1—carp, eel, perch, trout, pike, sardine, sole, herring, flounder, salmon, mackerel, tuna, halibut, plaice, pollock, cod; GR2—smoked fishes; GR3—herring in a creamy sauce, pickled herring and fish products in tins; #—fats, chocolate, gelatin; * the distribution was not parametric, as verified by the Shapiro-Wilk test.

**Table 3:** Daily iodine intake (µg) from all food groups in the subgroups of adolescents from northern Poland.

Iodine Intake Determinants		Iodine Intake (µg/d)		*p* Value **
		Mean ± Standard Deviation	Median (Min–Max) *	
Macroregions of northern Poland	N (*n* = 2418)	92.54 ± 100.98	65.63 (0.30–1176.20)	<0.001 ^(1)^
	N-W (*n* = 684)	107.72 ± 118.91	74.20 (0.41–985.30)	
Location of the school	C-S (*n* = 342)	98.36 ± 123.95	64.68 (0.41–1033.06)	0.80 ^(2)^
	Sc (*n* = 644)	97.77 ± 103.96	69.15 (0.30–985.300)	
	Mc (*n* = 2022)	94.47 ± 102.07	66.80 (0.41–1176.20)	
	Bc (*n* = 94)	104.53 ± 111.91	70.71 (10.72–761.87)	
Sex	Female (*n* = 1751)	94.30 ± 107.85	66.44 (0.30–1176.20)	0.10 ^(1)^
	Male (*n* = 1351)	97.94 ± 102.06	68.05 (0.41–981.40)	
Age (years)	14–17 (*n* = 2195)	96.63 ± 107.62	66.83 (0.30–1176.20)	0.80 ^(1)^
	18–20 (*n* = 907)	94.08 ± 99.73	67.18 (0.41–881.45)	
Body Mass Index classification	Uw (*n* = 156)	92.06 ± 85.01	68.30 (0.30–538.50)	0.76 ^(2)^
	Nw (*n* = 2178)	97.06 ± 107.51	67.42 (0.41–1176.20)	
	Ow (*n* = 465)	93.41 ± 101.01	66.78 (0.41–985.30)	
	Ob (*n* = 303)	93.22 ± 106.07	63.00 (9.20–1102.40)	
Iodine supplementation	Yes (*n* = 164)	103.62 ± 138.33	67.56 (0.71–1176.20)	0.90 ^(1)^
	No (*n* = 2938)	95.46 ± 103.23	66.83 (0.30–1102.40)	

N—Northern, N-W—North-Western; C-S—countryside, Sc—small city, Mc—medium city, Bc—big city; Uw—underweight, Nw—normal weight, Ow—overweight, Ob—obesity; * the distribution was not parametric, as verified by the Shapiro-Wilk test; ** comparisons were made using the ^(1)^ U Mann-Whitney test or ^(2)^ Kruskal-Wallis test; *p* ≤ 0.05.

**Table 4:** Correlations between iodine intake from different group products and total iodine intake, and between iodine-fortified salt intake and BMI and total iodine intake in a group of adolescents from northern Poland.

Variable		Correlations	
		R *	*p*
Iodine intake from different group products (µg/day) vs. total iodine intake (µg/day)	Dairy products	0.61	<0.001
	Eggs	0.37	<0.001
	Meat and meat products	0.39	<0.001
	Fish and fish products	0.15	<0.001
	Cereals	0.48	<0.001
	Vegetables, legumes, potatoes and fruits	0.54	<0.001
	Nuts and seeds	0.41	<0.001
	Beverages	0.51	<0.001
	Iodine-fortified salt	0.63	<0.001
	Other #	0.42	<0.001
Iodine-fortified salt intake (g/day) vs. total iodine intake (µg/day)		0.63	<0.001
Body Mass Index (kg/m^2^) vs. total iodine intake (µg/day)		−0.02	0.28

#—fats, chocolate, gelatin; * non-parametric Spearman test.

## Data Availability

The raw data supporting the conclusions of this article will be made available by the authors on request.

## Supplementary Materials

The following supporting information can be downloaded at: https://www.mdpi.com/article/10.3390/nu17243813/s1, Figure S1: Percentage of adolescents with dietary iodine intake below the recommended dietary allowance (RDA: <150 µg/day) level in different subgroups of adolescents from northern Poland; Figure S2: Percentage of adolescents with iodine-fortified salt intake: 0 g, (0, 5⟩ g and more than 5 g per day in different subgroups of adolescents from northern Poland; Table S1: Daily iodine intake from various food groups in the subgroups of adolescents from macroregions of northern Poland; Table S2: Daily iodine intake from different food groups in subgroups of adolescents by school location in northern Poland; Table S3: Daily iodine intake from different food groups in subgroups of female and male adolescents from northern Poland; Table S4: Daily iodine intake from different food groups in subgroups of adolescents by age of participants from northern Poland; Table S5: Daily iodine intake from different food groups in subgroups of adolescents from northern Poland by Body Mass Index (BMI) classification; Table S6: Daily iodine intake from different food groups in subgroups of adolescents from northern Poland by iodine supplementation.

## Abbreviations

The following abbreviations are used in this manuscript:

dIi	dietary iodine intake, intake of iodine from dietary sources
EAR	estimated average requirement
IDDs	iodine deficiency disorders
GR1	carp, eel, perch, trout, pike, sardine, sole, herring, flounder, salmon, mackerel, tuna, halibut, plaice, pollock, cod
GR2	smoked fishes
GR3	herring in a creamy sauce, pickled herring and fish products in tins
RDA	recommended dietary allowance
UL	tolerable upper intake level
