# Supplementary material for: Analysis of Determinants of Dietary Iodine Intake of Adolescents from Northern Regions of Poland: Coastal Areas and Lake Districts
Source: Nutrients. 2025 Dec 5;17(24):3813. doi: 10.3390/nu17243813 (PMC12736134; doi:10.3390/nu17243813)
Supplement: Supplementary file 1 [file nutrients-17-03813-s001.zip › nutrients-4002078-supplementary.pdf]

nutrients

MDPI

Supplementary Materials

Analysis of determinants of dietary iodine intake of adolescents from northern regions of Poland: coastal areas and lake districts

Katarzyna Lachowicz and Małgorzata Stachoń

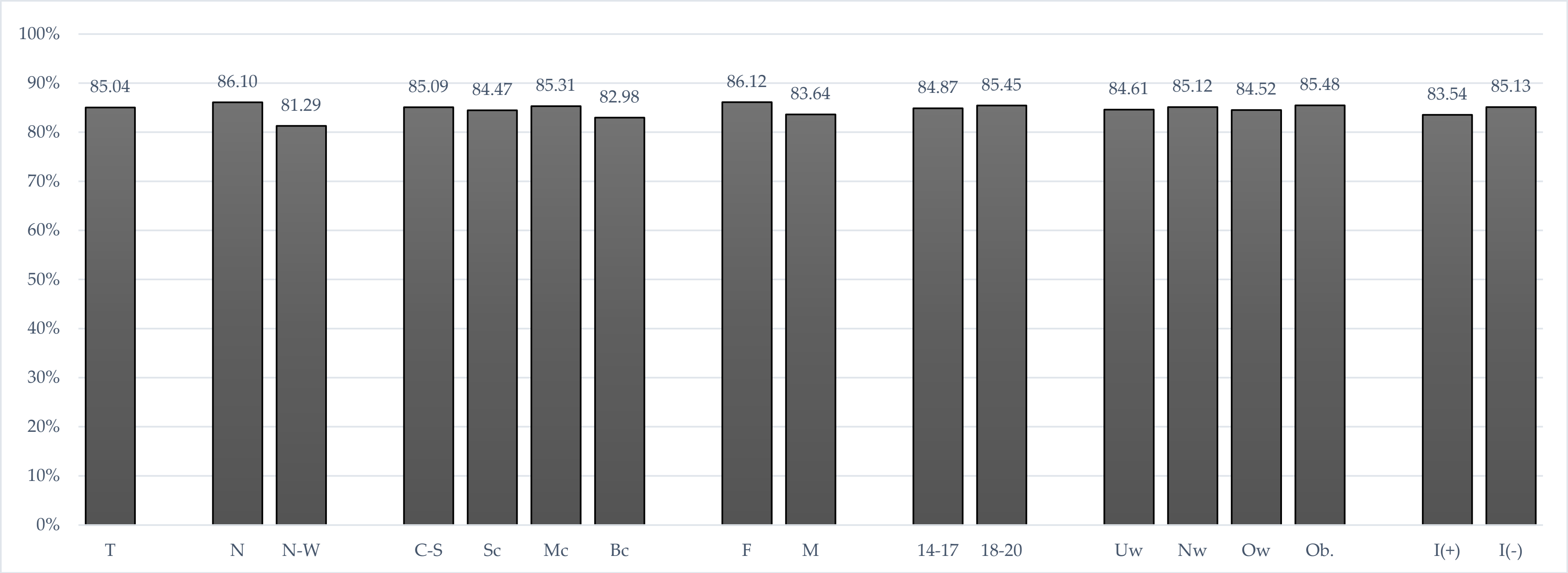

Figure S1. Percentage of adolescents with dietary iodine intake below the recommended dietary allowance (RDA: <150 µg/day) level in different subgroups of adolescents from northern Poland (T – total, macroregions: N – Northern, N-W – North-Western; location of the school: C-S – countryside, Sc – small city, Mc – medium city, Bc – big city; sex: F – female, M – male; age: 14-17 years old, 18-20 years old; Body Mass Index classification: Uw – underweight, Nw – normal weight, Ow – overweight, Ob. – obesity; iodine supplementation: I(+) – supplementing iodine, I(-) – not supplementing iodine).

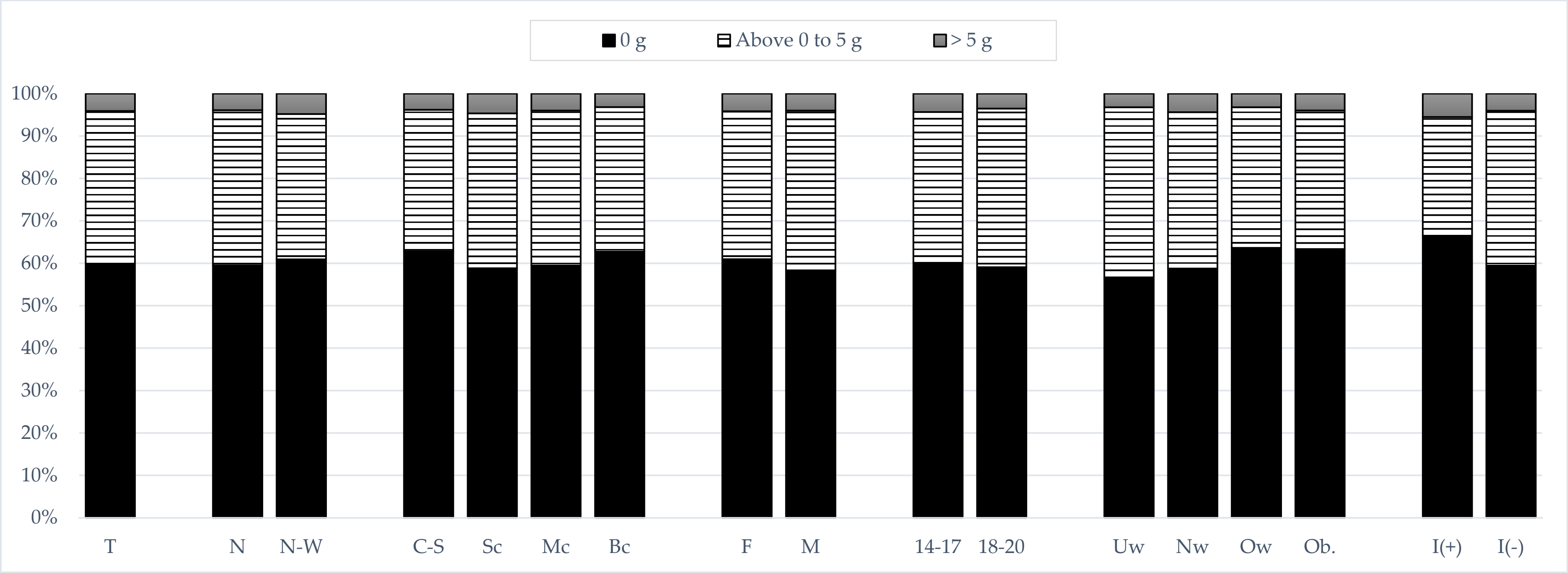

Figure S2. Percentage of adolescents with iodine-fortified salt intake: 0g, ⟨0,5⟩ g and more than 5 g per day in different subgroups of adolescents from northern Poland (T – total; macroregions of Poland: N – Northern, N-W – North-Western; location of the school: C-S – countryside, Sc – small city, Mc – medium city, Bc – big city; sex: F – female, M – male; age: 14-17 years old, 18-20 years old; Body Mass Index classification: Uw – underweight, Nw – normal weight, Ow – overweight, Ob. – obesity; iodine supplementation: I(+) – supplementing iodine, I(-) – not supplementing iodine).

Table S1. Daily iodine intake from various food groups in the subgroups of adolescents from macroregions of northern Poland.

Iodine source		Intake (μg)	Macroregions of northern Poland N (n=2418)	N-W (n=684)	*p* Value **
Dairy products		Mean±SD	20.92±23.42	25.41±23.69	**<0.0001**
		Median* (Min-Max)	14.34 (0.00-283.20)	18.46 (0.00-186.19)	
Eggs		Mean±SD	2.58±2.88	0.00±0.00	**0.0078**
		Median* (Min-Max)	2.01 (0.00-26.86)	2.01 (0.00-26.85)	
Meat and meat products		Mean±SD	2.72±2.67	4.20±3.60	**<0.0001**
		Median* (Min-Max)	2.01 (0.00-22.43)	3.09 (0.00-19.91)	
Fish and fish products	Total	Mean±SD	5.85±10.10	5.48±7.65	0.1513
		Median* (Min-Max)	2.25 (0.00-95.42)	2.59 (0.00-59.58)	
	GR1	Mean±SD	4.28±8.08	4.01±6.41	0.0591
		Median* (Min-Max)	1.57 (0.00-81.79)	1.63 (0.00-59.58)	
	GR2	Mean±SD	1.06±2.49	0.88±1.86	0.6749
		Median* (Min-Max)	0.00 (0.00-33.30)	0.00 (0.00-29.97)	
	GR3	Mean±SD	0.48±1.11	0.47±1.40	0.3772
		Median* (Min-Max)	0.00 (0.00-16.19)	0.00 (0.00-16.97)	
Cereals		Mean±SD	2.76±2.70	3.61±3.43	**<0.0001**
		Median* (Min-Max)	1.98 (0.00-26.88)	2.59 (0.00-21.81)	
Vegetables, legumes, potatoes and fruits		Mean±SD	11.08±11.94	11.26±12.13	0.6298
		Median* (Min-Max)	7.94 (0.30-155.87)	7.96 (0.00-113.42)	
Nuts and seeds		Mean±SD	4.44±7.26	5.62±8.78	**0.0015**
		Median* (Min-Max)	2.73 (0.00-65.29)	2.73 (0.00-69.39)	
Beverages		Mean±SD	18.24±63.94	23.44±82.44	0.5549
		Median* (Min-Max)	5.43 (0.00-807.14)	5.71 (0.00-789.30)	
Iodine-fortified salt		Mean±SD	23.28±49.58	24.41±50.34	0.8794
		Median* (Min-Max)	0.00 (0.00-556.63)	0.00 (0.00-458.40)	
Other#		Mean±SD	0.67±0.98	0.90±1.15	**<0.0001**
		Median* (Min-Max)	0.43 (0.00-12.71)	0.58 (0.00-11.92)	

GR1 – carp, eel, perch, trout, pike, sardine, sole, herring, flounder, salmon, mackerel, tuna, hali-but, plaice, pollock, cod; GR2 – smoked fishes; GR3 – herring in a creamy sauce, pickled herring and fish products in tins; # - fats, chocolate, gelatin; macroregions of northern Poland: N – Northern, N-W – North-Western; * the distribution was not parametric, as verified by the Shapiro-Wilk test; ** comparisons were made using the U Mann-Whitney test; $p \leq 0.05$ in bold.

Table S2. Daily iodine intake from different food groups in subgroups of adolescents by school location in northern Poland.

**Iodine source**		**Intake (μg)**	**Location of the school**				***p* Value ****
			**C-S (n=342)**	**Sc (n=644)**	**Mc (n=2022)**	**Bc (n=94)**	
Dairy products		Mean±SD	22.42±24.25	22.65±27.84	21.61±22.12	21.65±20.69	0.8379
		Median* (Min-Max)	15.83 (0.00-196.34)	14.45 (0.00-283.24)	15.04 (0.00-218.00)	14.38 (0.00-96.24)	
Eggs		Mean±SD	2.80±3.68	2.87±3.60	2.70±3.10	2.90±2.95	0.7819
		Median* (Min-Max)	2.01 (0.00-26.86)	2.01 (0.00-26.86)	2.01 (0.00-26.86)	2.01 (0.00-13.43)	
Meat and meat products		Mean±SD	3.14±3.16	3.11±3.03	2.97±2.90	3.85±3.07	**0.0104**
		Median* (Min-Max)	2.24[A] (0.00-17.73)	2.24[A] (0.00-19.64)	2.15[A] (0.00-22.43)	3.22[B] (0.00-16.16)	
Fish and fish prod-ucts	Total	Mean±SD	6.08±10.03	6.21±11.26	5.58±9.06	5.66±6.98	0.2333
		Median* (Min-Max)	2.65 (0.00-79.62)	2.61 (0.00-95.42)	2.12 (0.00-78.58)	2.98 (0.00-38.39)	
	GR1	Mean±SD	4.27±7.71	4.70±9.60	4.07±7.12	4.53±6.23	0.2425
		Median* (Min-Max)	1.62 (0.00-67.78)	1.62 (0.00-81.79)	1.57 (0.00-69.42)	2.38 (0.00-38.26)	
	GR2	Mean±SD	1.19±2.66	0.97±2.89	1.01±2.35	0.87±1.84	0.6985
		Median* (Min-Max)	0.00 (0.00-28.80)	0.00 (0.00-29.97)	0.00 (0.00-33.30)	0.00 (0.00-11.10)	
	GR3	Mean±SD	0.59±1.46	0.51±1.35	0.46±1.08	0.24±0.52	0.1784
		Median* (Min-Max)	0.00 (0.00-16.97)	0.00 (0.00-15.93)	0.00 (0.00-16.19)	0.00 (0.00-2.39)	
Cereals		Mean±SD	2.77±2.63	2.85±2.87	3.00±2.96	3.22±2.58	0.1227
		Median* (Min-Max)	2.03 (0.00-17.67)	2.06 (0.00-25.80)	2.11 (0.00-26.88)	2.40 (0.00-11.44)	
Vegetables, legumes, po-tatoes and fruits		Mean±SD	10.66±9.66	11.60±14.27	11.11±11.67	9.56±8.46	0.2993
		Median* (Min-Max)	8.46 (0.30-106.37)	7.34 (0.30-155.87)	8.11 (0.00-153.26)	6.64 (0.00-43.26)	
Nuts and seeds		Mean±SD	5.26±9.42	4.91±7.51	4.53±7.33	4.67±7.65	0.8505
		Median* (Min-Max)	2.33 (0.00-68.21)	2.73 (0.00-54.57)	2.73 (0.00-69.39)	2.54 (0.00-53.99)	
Beverages		Mean±SD	23.85±93.79	17.69±56.66	18.65±65.11	30.66±97.55	0.1767
		Median* (Min-Max)	4.64 (0.00-807.14)	6.14 (0.00-778.57)	5.43 (0.00-789.29)	7.86 (0.00-748.57)	
Iodine-fortified salt		Mean±SD	20.63±47.11	25.14±51.03	23.60±50.25	21.60±37.75	0.4317
		Median* (Min-Max)	0.00 (0.00-491.14)	0.00 (0.00-491.14)	0.00 (0.00-556.63)	0.00 (0.00-163.71)	

Other#	Mean±SD<br>Median*<br>(Min-Max)	0.75±1.04<br>0.47^AB<br>(0.04-11.92)	0.74±1.00<br>0.49^AB<br>(0.00-11.71)	0.71±1.04<br>0.44^A<br>(0.00-12.71)	0.77±0.66<br>0.55^B<br>(0.00-3.38)	**0.0222**

GR1 – carp, eel, perch, trout, pike, sardine, sole, herring, flounder, salmon, mackerel, tuna, halibut, plaice, pollock, cod; GR2 – smoked fishes; GR3 – herring in a creamy sauce, pickled herring and fish products in tins; # - fats, chocolate, gelatin; C-S – countryside, Sc – small city, Mc – medium city, Bc – big city; * the distribution was not parametric, as verified by the Shapiro-Wilk test; ** comparisons were made using Kruskal-Wallis test; values marked with different letters (A, B) in the rows are significantly different; $p \leq 0.05$ in bold.

Table S3. Daily iodine intake from different food groups in subgroups of female and male adolescents from northern Poland.

Iodine source		Intake (µg)	Sex: Female (n=1751)	Sex: Male (n=1351)	*p* Value **
Dairy products		Mean±SD<br>Median*<br>(Min-Max)	21.53±22.18<br>15.05<br>(0.00-217.99)	22.34±25.35<br>15.01<br>(0.00-283.24)	0.9758
Eggs		Mean±SD<br>Median*<br>(Min-Max)	2.69±3.12<br>2.01<br>(0.00-26.86)	2.84±3.46<br>2.01<br>(0.00-26.86	0.6349
Meat and meat products		Mean±SD<br>Median*<br>(Min-Max)	2.90±2.86<br>2.13<br>(0.00-19.64)	3.23±3.09<br>2.37<br>(0.00-22.43)	**0.0015**
Fish and fish products	Total	Mean±SD<br>Median*<br>(Min-Max)	5.62±9.39<br>2.17<br>(0.00-96.42)	5.95±9.90<br>2.51<br>(0.00-83.44)	0.4876
	GR1	Mean±SD<br>Median*<br>(Min-Max)	4.13±7.50<br>1.57<br>(0.00-81.79)	4.39±8.05<br>1.62<br>(0.00-69.42)	0.8064
	GR2	Mean±SD<br>Median*<br>(Min-Max)	1.00±2.32<br>0.00<br>(0.00-33.30)	1.04±2.42<br>0.00<br>(0.00-29.97)	0.7046
	GR3	Mean±SD<br>Median*<br>(Min-Max)	0.47±1.27<br>0.00<br>(0.00-16.97)	0.49±1.05<br>0.00<br>(0.00-9.56)	**0.0441**
Cereals		Mean±SD<br>Median*<br>(Min-Max)	2.89±2.81<br>2.04<br>(0.00-25.80)	3.03±3.00<br>2.17<br>(0.00-26.88)	0.1939
Vegetables, legumes, potatoes and fruits		Mean±SD<br>Median*<br>(Min-Max)	11.02±11.22<br>8.16<br>(0.00-113.40)	11.24±12.90<br>7.81<br>(0.00-155.87)	0.3835
Nuts and seeds		Mean±SD	4.50±7.12	4.95±8.25	0.9244

	Median* (Min-Max)	2.73 (0.00-63.34)	2.73 (0.00-69.39)	
Beverages	Mean±SD	19.54±70.46	19.19±65.82	0.5664
	Median* (Min-Max)	5.36 (0.00-807.14)	5.71 (0.00-779.29)	
Iodine-fortified salt	Mean±SD	22.85±50.14	0.00±0.00	0.1130
	Median* (Min-Max)	0.00 (0.00-556.63)	0.00 (0.00-491.14)	
Other#	Mean±SD	0.70±0.95	0.75±1.11	0.2995
	Median* (Min-Max)	0.44 (0.00-12.71)	0.46 (0.00-11.92)	

GR1 – carp, eel, perch, trout, pike, sardine, sole, herring, flounder, salmon, mackerel, tuna, halibut, plaice, pollock, cod; GR2 – smoked fishes; GR3 – herring in a creamy sauce, pickled herring and fish products in tins; # - fats, chocolate, gelatin; C-S – countryside, Sc – small city, Mc – medium city, Bc – big city; * the distribution was not parametric, as verified by the Shapiro-Wilk test; ** comparisons were made using the U Mann-Whitney test; $p \leq 0.05$ in bold.

Table S4. Daily iodine intake from different food groups in subgroups of adolescents by age of participants from northern Poland.

Iodine source		Intake (µg)	Age 14-17 years old (n=2195)	Age 18-20 years old (n=907)	*p* Value **
Dairy products		Mean±SD	21.95±23.45	21.83±24.02	0.9089
		Median* (Min-Max)	15.24 (0.00-271.71)	14.40 (0.00-283.24)	
Eggs		Mean±SD	2.77±3.32	2.71±3.17	0.6820
		Median* (Min-Max)	2.01 (0.00-26.86)	2.01 (0.00-26.86)	
Meat and meat products		Mean±SD	3.04±3.00	3.06±2.90	0.4854
		Median* (Min-Max)	2.21 (0.00-22.43)	2.24 (0.00-19.00)	
Fish and fish products	Total	Mean±SD	5.68±9.42	5.98±10.08	0.1563
		Median* (Min-Max)	2.19 (0.00-83.44)	2.92 (0.00-95.42)	
	GR1	Mean±SD	4.17±7.43	4.41±8.45	0.4126
		Median* (Min-Max)	1.57 (0.00-69.42)	1.62 (0.00-81.79)	
	GR2	Mean±SD	1.01±2.39	1.03±2.28	0.6488
		Median* (Min-Max)	0.00 (0.00-33.30)	0.00 (0.00-23.67)	
	GR3	Mean±SD	0.46±1.13	0.50±1.29	0.2183
		Median* (Min-Max)	0.00 (0.00-15.93)	0.00 (0.00-16.97)	

Cereals	Mean±SD	2.97±2.93	2.91±2.81	0.8787
	Median* (Min-Max)	2.10 (0.00-26.88)	2.10 (0.00-25.80)	
Vegetables, legumes, potatoes and fruits	Mean±SD	11.03±11.03	11.33±14.02	0.1236
	Median* (Min-Max)	8.10 (0.00-138.50)	7.56 (0.00-155.87)	
Nuts and seeds	Mean±SD	4.65±7.59	4.82±7.74	0.7753
	Median* (Min-Max)	2.73 (0.00-69.39)	2.73 (0.00-54.57)	
Beverages	Mean±SD	19.96±71.06	18.01±61.78	0.1548
	Median* (Min-Max)	5.71 (0.00-807.14)	5.21 (0.00-779.29)	
Iodine-fortified salt	Mean±SD	23.89±50.06	22.67±47.62	0.8267
	Median* (Min-Max)	0.00 (0.00-556.63)	0.00 (0.00-491.14)	
Other#	Mean±SD	0.71±0.97	0.75±1.13	0.8191
	Median* (Min-Max)	0.46 (0.00-12.71)	0.45 (0.00-11.92)	

GR1 – carp, eel, perch, trout, pike, sardine, sole, herring, flounder, salmon, mackerel, tuna, halibut, plaice, pollock, cod; GR2 – smoked fishes; GR3 – herring in a creamy sauce, pickled herring and fish products in tins; # - fats, chocolate, gelatin; C-S – countryside, Sc – small city, Mc – medium city, Bc – big city; * the distribution was not parametric, as verified by the Shapiro-Wilk test; ** comparisons were made using the U Mann-Whitney test; $p \leq 0.05$.

Table S5. Daily iodine intake from different food groups in subgroups of adolescents from northern Poland by Body Mass Index (BMI) classification.

Iodine source		Intake (μg)	Body Mass Index classification				*p* Value **
			Uw (n=156)	Nw (n=2178)	Ow (n=465)	Ob (n=303)	
Dairy products		Mean±SD	19.53±20.71	21.70±22.37	22.74±27.39	23.44±27.15	0.6740
		Median* (Min-Max)	14.26 (0.00-185.67)	14.94 (0.00-210.04)	15.43 (0.00-283.24)	15.86 (0.00-218.00)	
Eggs		Mean±SD	2.22±2.43	2.73±3.18	0.00±0.00	0.00±0.00	0.5924
		Median* (Min-Max)	2.01 (0.00-24.17)	2.01 (0.00-26.86)	2.01 (0.00-26.86)	2.01 (0.00-22.83)	
Meat and meat products		Mean±SD	2.70±2.87	3.08±2.94	2.92±2.97	3.19±3.16	0.0748
		Median* (Min-Max)	1.84 (0.00-17.73)	2.24 (0.00-22.43)	1.99 (0.00-17.47)	2.43 (0.00-19.64)	
Fish and fish products	Total	Mean±SD	6.68±11.24	5.75±9.51	5.51±9.66	5.82±9.42	0.9061
		Median* (Min-Max)	2.91 (0.00-64.26)	2.41 (0.00-95.42)	2.06 (0.00-78.58)	1.90 (0.00-83.44)	
	GR1	Mean±SD	4.87±8.89	4.26±7.80	3.93±7.20	4.27±7.47	0.8902

	Median* (Min-Max)	2.07 (0.00-57.79)	1.62 (0.00-81.79)	1.57 (0.00-66.45)	1.57 (0.00-67.00)	
GR2	Mean±SD	1.17±2.44	1.00±2.30	1.08±2.80	1.01±1.99	0.9061
	Median* (Min-Max)	0.00 (0.00-11.78)	0.00 (0.00-33.30)	0.00 (0.00-23.67)	0.00 (0.00-13.32)	
GR3	Mean±SD	0.59±1.83	0.47±1.10	0.46±1.28	0.51±1.17	0.6778
	Median* (Min-Max)	0.00 (0.00-16.97)	0.00 (0.00-15.93)	0.00 (0.00-16.19)	0.00 (0.00-7.67)	
Cereals	Mean±SD	2.45±1.90	2.99±2.90	2.90±2.84	3.00±3.37	0.3221
	Median* (Min-Max)	1.94 (0.00-9.36)	2.11 (0.00-25.26)	2.17 (0.00-20.12)	2.03 (0.00-26.88)	
Vegetables, legumes, potatoes and fruits	Mean±SD	11.74±8.91	11.01±11.72	11.13±13.29	11.51±13.08	0.1336
	Median* (Min-Max)	9.21 (0.30-46.81)	7.91 (0.00-153.30)	7.59 (0.30-159.87)	8.01 (0.30-13.85)	
Nuts and seeds	Mean±SD	4.60±6.66	4.56±7.41	5.25±7.99	4.85±8.99	0.2731
	Median* (Min-Max)	2.73 (0.00-42.83)	2.73 (0.00-69.39)	2.73 (0.00-54.57)	2.14 (0.00-68.21)	
Beverages	Mean±SD	12.56±35.58	20.58±71.30	17.22±65.31	17.61±65.18	0.1224
	Median* (Min-Max)	4.21 (0.00-400.00)	5.71 (0.00-807.14)	5.43 (0.00-778.57)	5.36 (0.00-773.21)	
Iodine-fortified salt	Mean±SD	28.86±61.20	23.93±50.17	21.97±47.09	20.31±43.66	0.1486
	Median* (Min-Max)	0.00 (0.00-491.14)	0.00 (0.00-556.63)	0.00 (0.00-491.14)	0.00 (0.00-327.43)	
Other#	Mean±SD	0.70±1.25	0.72±0.95	0.78±1.35	0.66±0.75	0.5480
	Median* (Min-Max)	0.41 (0.00-11.43)	0.46 (0.00-12.71)	0.45 (0.00-11.92)	0.44 (0.06-4.89)	

GR1 – carp, eel, perch, trout, pike, sardine, sole, herring, flounder, salmon, mackerel, tuna, halibut, plaice, pollock, cod; GR2 – smoked fishes; GR3 – herring in a creamy sauce, pickled herring and fish products in tins; # - fats, chocolate, gelatin; C-S – countryside, Sc – small city, Mc – medium city, Bc – big city; * the distribution was not parametric, as verified by the Shapiro-Wilk test; ** comparisons were made using Kruskal-Wallis test; values marked with different letters (A, B, C) in the rows are significantly different; $p \leq 0.05$.

Table S6. Daily iodine intake from different food groups in subgroups of adolescents from northern Poland by iodine supplementation.

Iodine source	Intake (µg)	Iodine supplementation Yes (n=164)	No (n=2938)	*p* Value **
Dairy products	Mean±SD	21.73±22.4	21.83±23.68	0.7825
	Median* (Min-Max)	14.78 (0.00-140.20)	15.04 (0.00-283.24)	
Eggs	Mean±SD	3.15±3.47	2.73±3.26	**0.0233**
	Median* (Min-Max)	2.69 (0.00-20.14)	2.01 (0.00-26.86)	
Meat and meat products	Mean±SD	4.03±3.10	2.99±2.95	**<0.0001**
	Median* (Min-Max)	3.36 (0.00-15.14)	2.16 (0.00-22.43)	

Fish and fish products	Total	Mean±SD	6.20±8.52	5.74±9.67	0.2412
		Median* (Min-Max)	3.35 (0.00-57.60)	2.38 (0.00-95.42)	
	GR1	Mean±SD	4.55±6.92	4.22±7.78	0.3103
		Median* (Min-Max)	1.86 (0.00-48.04)	1.62 (0.00-81.79)	
	GR2	Mean±SD	1.05±1.91	1.02±2.38	0.6954
		Median* (Min-Max)	0.00 (0.00-11.10)	0.00 (0.00-33.30)	
	GR3	Mean±SD	0.56±1.23	0.47±1.18	0.2393
		Median* (Min-Max)	0.00 (0.00-9.56)	0.00 (0.00-16.97)	
Cereals		Mean±SD	3.24±3.32	2.93±2.87	0.5050
		Median* (Min-Max)	2.17 (0.00-21.92)	2.10 (0.00-26.88)	
Vegetables, legumes, potatoes and fruits		Mean±SD	10.57±11.36	11.15±12.02	0.1639
		Median* (Min-Max)	6.74 (0.30-78.40)	7.99 (0.00-155.87)	
Nuts and seeds		Mean±SD	4.57±7.68	4.70±7.63	0.4251
		Median* (Min-Max)	2.43 (0.00-42.86)	2.73 (0.00-69.39)	
Beverages		Mean±SD	25.25±97.70	19.04±66.47	0.4898
		Median* (Min-Max)	5.11 (0.00-755.70)	5.64 (0.00-807.14)	
Iodine-fortified salt		Mean±SD	23.66±60.15	23.52±49.11	0.0723
		Median* (Min-Max)	0.00 (0.00-327.43)	0.00 (0.00-556.63)	
Other#		Mean±SD	0.94±1.14	0.71±1.01	**0.0016**
		Median* (Min-Max)	0.55 (0.00-7.75)	0.45 (0.00-12.71)	

GR1 – carp, eel, perch, trout, pike, sardine, sole, herring, flounder, salmon, mackerel, tuna, halibut, plaice, pollock, cod; GR2 – smoked fishes; GR3 – herring in a creamy sauce, pickled herring and fish products in tins; # - fats, chocolate, gelatin; C-S – countryside, Sc – small city, Mc – medium city, Bc – big city; * the distribution was not parametric, as verified by the Shapiro-Wilk test; ** comparisons were made using the U Mann-Whitney test; $p \leq 0.05$ in bold.
